# New calculations indicate that 90% of flowering plant species are animal-pollinated

**DOI:** 10.1093/nsr/nwad219

**Published:** 2023-08-11

**Authors:** Ze-Yu Tong, Ling-Yun Wu, Hui-Hui Feng, Meng Zhang, W Scott Armbruster, Susanne S Renner, Shuang-Quan Huang

**Affiliations:** Institute of Evolution and Ecology, School of Life Sciences, Central China Normal University, China; Institute of Evolution and Ecology, School of Life Sciences, Central China Normal University, China; Faculty of Resources and Environmental Science, Hubei University, China; Institute of Evolution and Ecology, School of Life Sciences, Central China Normal University, China; Institute of Evolution and Ecology, School of Life Sciences, Central China Normal University, China; School of Biological Sciences, University of Portsmouth, UK; Institute of Arctic Biology, University of Alaska Fairbanks, USA; Department of Biology, Washington University, USA; Institute of Evolution and Ecology, School of Life Sciences, Central China Normal University, China

The services of animal pollinators that deliver compatible pollen to receptive stigmas are essential for the reproduction of many flowering plants [1,2]. The maintenance of plant-pollinator interactions, therefore, is the basis for the function and sustainability of healthy ecosystems. For this reason, there has been widespread concern over the past two decades about the global decline in pollinators and the effects of this loss on pollination services for both wild plants and crops [3,4]. One fundamental question relevant to the ongoing pollinator loss remains only partially answered, namely: What proportion of the world's angiosperm species are pollinated by animals? The best estimate so far is that of Ollerton and his colleagues [5] who inferred that between 85% and 87.5% of the ca. 352 000 species of flowering plants are animal-pollinated. These proportions were obtained by combining mean values of animal-pollinated percentages of flowering plants reported in surveys of pollination systems in various communities. Together, these communities included 3918 plant species, ranging from 15 to 391 species per community ([5]: Appendix 1). The higher proportion (87.5%) reflects weighting by species diversity across the three latitudinal belts in which the investigated communities were located (tropical, subtropical, temperate), while the lower value (85%) reflects the unweighted mean.

We use a different approach to estimate the percentage of flowering plants that depend on animals for their pollination. Our estimate is based on publications on pollination modes and the insight that pollination by wind or water, that is, abiotic pollination, is correlated with a suite of floral traits that form the two best-established floral syndromes in pollination ecology [1]. We therefore first calculated the number of families, genera, and species that are wind or water pollinated, modes that can reliably be inferred from a species’ morphology, and we then treated the remaining angiosperms as relying on animals for pollination. Separately, we also re-analyzed Ollerton *et al.*’s [5] data.

When the published pollination data found in our literature search were plotted on the GBIF (Global Biodiversity Information Facility) taxon list, 105 of 465 families contain abiotically pollinated species, leaving 67% of families as animal-pollinated. Of 14 437 genera, 1573 are abiotically pollinated, and 88 include species with both biotic and abiotic pollination, which yielded 88% exclusively animal-pollinated genera [1−(1573 + 88)/14 437]. Of 332 341 angiosperm species in the GBIF database, 33 623 are abiotically pollinated, and 296 species utilize both biotic and abiotic pollination. The percentage of animal-pollinated flowering species is thus 90% [1−(33 623 + 296)/332 341] (Table S1; Fig. 1).

**Figure 1.**
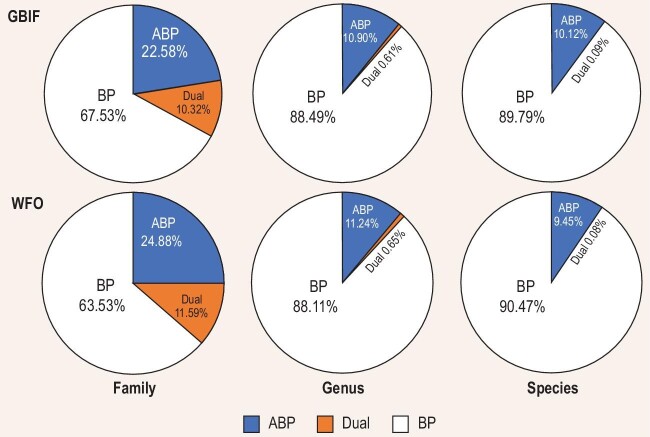
Percentages of biotic (BP), abiotic (ABP), and ambophilous (Dual) pollination based on the results of our literature search (Table S3) and the use of the most complete angiosperm name databases GBIF and WFO (accessed in February 2023). Table S4 shows the differences in family circumscription between GBIF, WFO and APG IV.

When the data were plotted on the WFO (World Flora Online) taxon list, 103 of 414 families are abiotically pollinated and 64% of families are animal-pollinated. Of 13 772 genera, 1548 are abiotically pollinated, and 90 include species with both biotic and abiotic pollination, which, based on our formula, yields 88% exclusively animal-pollinated genera. Of 339 876 angiosperm species in the WFO database, 32 129 are abiotically pollinated, and 267 species utilize both biotic and abiotic pollination. The percentage of animal-pollinated flowering species is thus 90% [1−(32 129 + 267)/339 876] (Table S2; Fig. 1). We also present the congruence between GBIF, WFO and APG IV (Angiosperm Phylogeny Group IV) in Table S3.

After extending and re-analyzing Ollerton *et al.*’s [5] data with sample-size weighting, the calculation of the global mean changed from 85% (in the original study) to 88.5% of angiosperms being biotically pollinated. After correcting for both sample size and latitudinal diversity patterns, the value changed from 87.5% to 89.5% of angiosperms being biotically pollinated.

Our finding that 90% of flowering-plant species are animal-pollinated depends on the corrected inference of the number of wind- or water-pollinated species and the assumed total number of angiosperm species. Neither of these two numbers will ever be final because of progress in plant discovery and field work, and because of changing species concepts. Recent estimates of the number of angiosperm species range from 295 383 to 369 434, in each case based on the best efforts of taxonomists at the Royal Botanic Gardens, Kew [6,7]. The total numbers of flowering plant species used in our study, namely 332 341 when using the GBIF or 339 876 when using the WFO, fall between these two values, and our results show that percentages resulting from these two data sources (namely 90%) are identical.

Thirty years ago, it was estimated that 23% (84 of 365) families and 8% (1106 of 13 500) genera are abiotically pollinated [8], while a slightly more recent estimate gave 20% of families as abiotically pollinated [9]. Our estimates are that 33%–36% of families and 11% of genera are pollinated by wind or water or are ambophilous. Our higher percentages of wind-pollinated families and genera may be explained by new insights on wind-pollinated genera as listed in Table S4 and perhaps also by our addition of ambophily to wind pollination. Even so, ambophily may be underestimated (supplementary discussion and Fig. S1).

A benchmark study on the question of which percentage of angiosperms might be animal-pollinated is that of Ollerton *et al.* [5], who used a community-level approach for calculation. Their communities included 3918 species, slightly more than 1% of the estimated number of angiosperms (352 000 at the time), which resulted in an estimate of 87.5% animal-pollinated angiosperms, while we inferred that 90% of the angiosperms rely on animal pollinators. Even closer correspondence between the results of Ollerton *et al.* [5] and our approach resulted when we extended and corrected their data, which yielded 89.5% of angiosperms as animal-pollinated. This degree of consilience suggests that the % of angiosperm species that are biotically pollinated is indeed about 90%. This fits nicely with the finding that insects have pollinated angiosperms for approximately 86% of this plant lineage's evolutionary history [10] and pinpoints the need for especially the conservation of insects.

## Supplementary Material

nwad219_Supplemental_Files
