# Supplementary material for: New calculations indicate that 90% of flowering plant species are animal-pollinated
Source: Natl Sci Rev. 2023 Aug 11;10(10):nwad219. doi: 10.1093/nsr/nwad219 (PMC10517183; doi:10.1093/nsr/nwad219)
Supplement: nwad219_Supplemental_Files [file nwad219_supplemental_files.zip › supplementary file.docx]

**Legends for online supporting supplemental figures and tables.**

**Online detailed Methods**

**Figure S1.** Examples of species that may be ambophilous and that require field experiments to resolve the predominant pollen vector in their natural habitat. (A) The pendulous inflorescences (spikes) of *Tetracentron sinense* (Trochodendraceae) suggest wind pollination, but the stamens’ filaments produce nectar drops (arrow in the insert panel in A). (B) Honeybee collecting nectar and pollen from the catkins of *Salix matsudana* (Salicaceae). The inset shows the sticky pollen attached to a dehisced anther. (C) A lavatory fly feeding on the liquid offered by the male flowers of *Castanea mollissima* (Fagaceae). (D) A mosquito collecting nectar from a green female flower of *Helwingia japonica* (Helwingiaceae); its head carries white pollen from a previously visited male plant. (E) An inflorescence of the dioecious *Sargentodoxa cuneata* (Lardizabalaceae); the inset shows the yellow greenish petals (white arrow). (F) Female flowers of the gynodioecious *Thalictrum smithii* (Ranunculaceae) without sepals, typical of wind pollination. (G) A bumblebee collecting pollen from a hermaphroditic flower of *Thalictrum delavayi*. (H) A halictid bee collecting pollen from white-flowered *Juncus allioides* (Juncaceae); the inset shows pollen tetrads (arrow) germinating on a stigma. (I) Sticky pollen grains presented on the dehisced anthers of *Paris polyphylla* (Melanthiaceae). Photo credits: Shuang-Quan Huang.

**Additional Discussion**

**References**

**Table S1.** Number of families, genera, and species (using the GBIF February 2023 family circumscriptions as shown in Table S4) with abiotic pollination (ABP) or ambophilous ‘Dual’) pollination.

**Table S2.** Number of families, genera, and species (using the WFO February 2023 family circumscriptions as shown in Table S4), with abiotic pollination (ABP) or ambophilous (‘Dual’) pollination.

**Table S3.** The 416, 465, and 414 families accepted by APG IV (2016) and the GBIF and WFO databases (both accessed in February 2023). Empty cells indicate absence of that family.

**Table S4.** Taxa with abiotic pollination (ABP), biotic pollination (BP), and both pollination modes (Dual) at the family, genus, and species level, based on the cited references. The rightmost column contains notes. See Table S3 for the different family concepts in APG (2016) and GBIF and WFO accessed in February 2023.
