# Supplementary material for: New calculations indicate that 90% of flowering plant species are animal-pollinated
Source: Natl Sci Rev. 2023 Aug 11;10(10):nwad219. doi: 10.1093/nsr/nwad219 (PMC10517183; doi:10.1093/nsr/nwad219)
Supplement: nwad219_Supplemental_Files [file nwad219_supplemental_files.zip › OSM_for_a_perspective_of_animal_pollinated_percent_SQHuang.docx]

**Online Supplementary Information for a perspective entitled “New calculations imply that 90% of flowering plant species are animal-pollinated”**

Ze-Yu Tong^1^, Ling-Yun Wu^1,2^, Hui-Hui Feng^1^, Meng Zhang^1^, W. Scott Armbruster^3^, Susanne S. Renner^4*^ & Shuang-Quan Huang^1*^

This manuscript has one figure in the main text, and online supplemental materials including detailed Methods, Figure S1 and additional Discussion in a **word** file, and Tables S1, S2, S3, and S4 presenting raw data as **Excel** files.

**ONLINE METHODS**

**Literature search for abiotic and ambophilous (‘dual’) pollination and use of two angiosperm taxon databases**

Wind pollination correlates with inflorescences with numerous small flowers, absent or minute petals, white or green flower colours, absence of nectar, absences of scent, feathery styles, a single ovule per flower, abundant pollen, dry (not sticky) pollen, protogyny, unisexual flowers, and other traits related to plant architecture and flowering times (Friedman and Barrett 2009). Water pollination is characterized by adaptations for the transport of pollen, or entire male flowers, on or in water (Cox 1988). Even so, ambophily may be underestimated (Abrahamczyk et al. 2023, see the main text for the argue).

We conducted a literature search on abiotic pollination, by using ISI web of knowledge, setting the time window from 1900 to 3 March 2021. The keyword “abiotic pollinat*” generated 545 results, “water pollinat*” 2017 results, “wind pollinat*” 2032 results, “anemophily*” 165 results, “hydrophil*” 159 results, “ambophil*” 42 results, involving over 5000 publications (including books). We removed publications not focused on pollination (e.g., studies of wind-pollinated plants for reforestation; wind-pollinated *Zea mays* for physiological research; hydrating compounds in chemistry, etc.), leaving only taxa shown or inferred to be abiotically pollinated. In addition, we relied on compilations of water-pollinated species by Cox (1988) and of ambophilous species, that is species pollinated by both insects and wind, by Abrahamczyk et al. (2023). All species and genera with published information on their pollination were then found and scored in two databases of angiosperm taxa, namely the Global Biodiversity Information Facility (GBIF, https://www.gbif.org, accessed on 22 February 2023 and the World Flora Online (WFO, http://www.worldfloraonline.org, accessed on the same day). Different family circumscriptions used by the Angiosperm Phylogeny Group classification (APG IV; Chase et al. 2016), and the GBIF and WFO databases are shown in Table S3. The raw data for how pollination modes were scored are presented in Supplementary Table S4. We have made Table S4 available online (http://sky.ccnu.edu.cn/info/1232/9914.htm) for use by the research community and for future updates.

**Calculation of biotic pollination in angiosperms**

The number of angiosperm species is currently unknown and will never be precisely known because of different species concepts, especially in economically important taxa with numerous forms of interest to farmers, and in groups with apomictic reproduction (of which there are hundreds) in which the biological species concept does not apply. An estimate of 352,000 species, made by taxonomists at the Royal Botanic Gardens, Kew (Paton et al. 2008), was used by Ollerton et al. (2011) in their calculation. The same 2008 species are also accepted in GBIF and WFO, but with newly described, newly accepted, or previously overlooked species added. Over the past 25 years, the numbers of accepted genera and families has increased greatly because of attempts to arrive at more monophyletic units, often based on molecular data. After pruning fossil angiosperm families (Archaefructaceae, Montsechiaceae) and modifying low-quality records (i.e., broken records, typos in taxon names), we found 465 families, 14,437 genera and 332,341 angiosperm species in GBIF, and 414 families, 13,772 genera, and 339,876 species in WFO.

We then calculated the percentage of angiosperms biotically pollinated by using Formula 1: [1- ((number of taxa with abiotic pollination + number of ambophilous taxa)/ total number of taxa)] × 100%

To re-analyse the data of Ollerton et al. (2011) who used sample-size weighting, where the size of the plant community (i.e., the number of observed species) was treated as the sample size, we first extended their dataset by obtaining approximate community (sample) sizes for the Moldenke and Lincoln (1979) plot in montane Colorado included in Ollerton et al.’s study and then used Table 2 of Ollerton et al. (2011) to estimate original community sizes by dividing the relevant species counts for the largest category by the percent of the flora that the species count represents, then scaling to 100 when the sum of percent values exceeded 100. Following Ollerton et al., we corrected for latitudinal trends in angiosperm diversity from the equator to the pole, using their global species estimate of 352,000 species (Paton et al. 2008). The tropical belt was estimated to contain 50.5% of global angiosperm diversity, the subtropical belt 27.7%, and the temperate belt 21.8% (see Kier et al. 2005; Ollerton et al. 2011). These proportions were then used to weight the latitudinal-zone contributions to the global mean estimate of the proportion of species biotically pollinated.

**References**

Abrahamczyk S, Struck JH, Weigend M. 2023. The best of two worlds: ecology and evolution of ambophilous plants. Biological Reviews 98: 391-420.

Chase MW, Christenhusz MJM, Fay MF, Byng JW, Judd WS, Soltis DE, Mabberley DJ, Sennikov AN, Soltis PS, Stevens PF. 2016. An update of the Angiosperm Phylogeny Group classification for the orders and families of flowering plants: APG IV. Botanical Journal of the Linnean Society 181: 1-20.

Cox PA. 1988. Hydrophilous pollination. Annual Review of Ecology and Systematics 19: 261-280.

Friedman J, Barrett SCH. 2009. Wind of change: new insights on the ecology and evolution of pollination and mating in wind-pollinated plants. Annals of Botany 103: 1515-1527.

Kier G, Mutke J, Dinerstein E, Ricketts TH, Küper W, Kreft H, Barthlott W. 2005. Global patterns of plant diversity and floristic knowledge. Journal of Biogeography 32: 1107-1116.

Moldenke AR, Lincoln PG. 1979. Pollination ecology in montane Colorado: a community analysis. Phytologia 42: 349-379

Ollerton J, Winfree R, Tarrant S. 2011. How many flowering plants are pollinated by animals? Oikos 120: 321-326.

Paton AJ, Brummitt N, Govaerts R, Harman K, Hinchcliffe S, Allkin B, Lughadha EN. 2008. Towards Target 1 of the Global Strategy for Plant Conservation: a working list of all known plant species—progress and prospects. Taxon 57: 602-611.

**Figure S1**


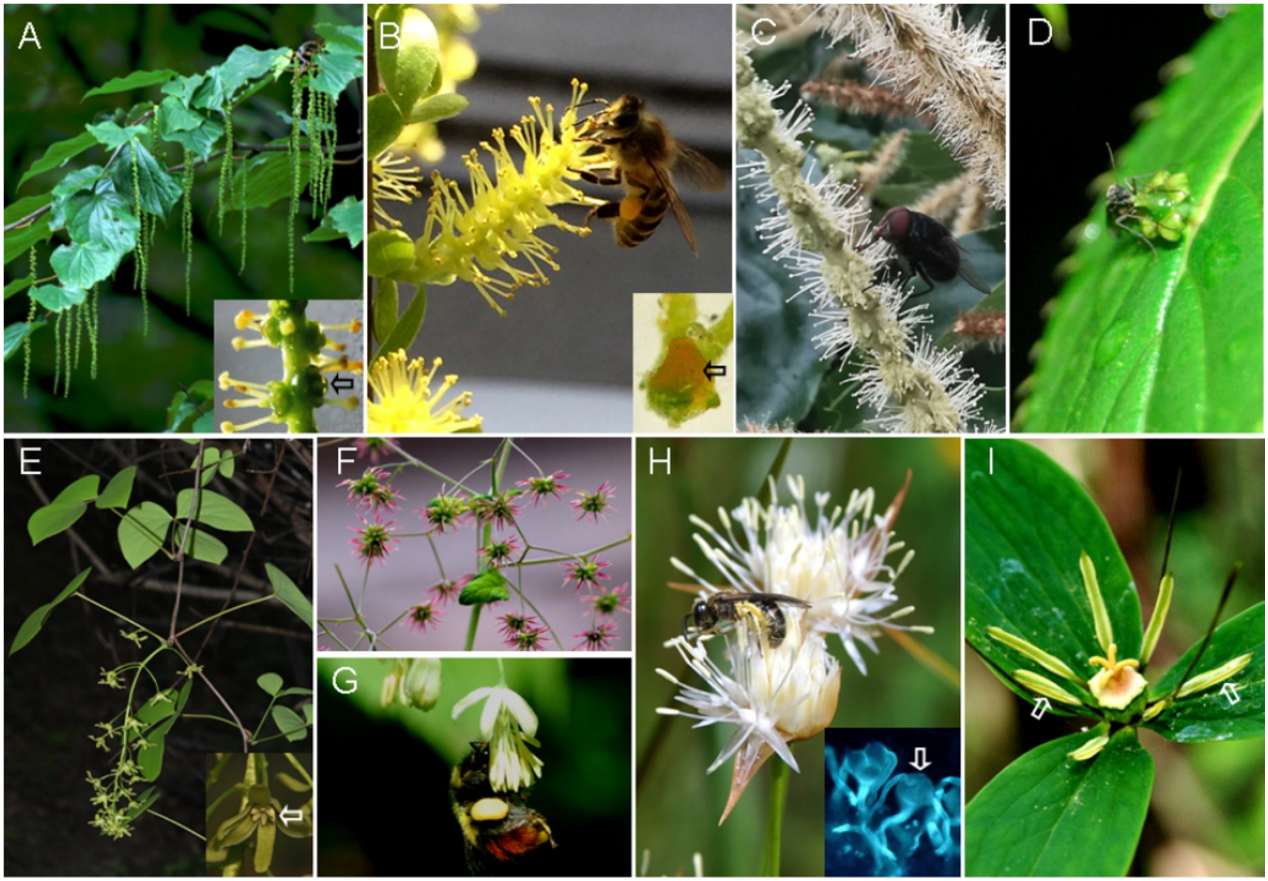


**Figure S1.** Examples of species that may be ambophilous and that require field experiments to resolve the predominant pollen vector in their natural habitat. (A) The pendulous inflorescences (spikes) of *Tetracentron sinense* (Trochodendraceae) suggest wind pollination, but the stamens’ filaments produce nectar drops (arrow in the insert panel in A). (B) Honeybee collecting nectar and pollen from the catkins of *Salix matsudana* (Salicaceae). The inset shows the sticky pollen attached to a dehisced anther. (C) A lavatory fly feeding on the liquid offered by the male flowers of *Castanea mollissima* (Fagaceae). (D) A mosquito collecting nectar from a green female flower of *Helwingia japonica* (Helwingiaceae); its head carries white pollen from a previously visited male plant. (E) An inflorescence of the dioecious *Sargentodoxa cuneata* (Lardizabalaceae); the inset shows the yellow greenish petals (white arrow). (F) Female flowers of the gynodioecious *Thalictrum smithii* (Ranunculaceae) without sepals, typical of wind pollination. (G) A bumblebee collecting pollen from a hermaphroditic flower of *Thalictrum delavayi*. (H) A halictid bee collecting pollen from white-flowered *Juncus allioides* (Juncaceae); the inset shows pollen tetrads (arrow) germinating on a stigma. (I) Sticky pollen grains presented on the dehisced anthers of *Paris polyphylla* (Melanthiaceae). Photo credits: Shuang-Quan Huang

**ONLINE DISCUSSIONS**

**Examples of ambophily and of the difficulties when relying on the pollination syndrome approach for inferring wind pollination**

The south Asian *Tetracentron sinense*, the sole species of this genus, which has

pendulous spikes with many small greenish flowers were thought to be wind-pollinated (Cronquist 1981), but field observations showed this species is ambophilous and can also be pollinated by syrphid flies and bees (Gan et al. 2013; authors’ unpublished data), matching its floral nectaries (Endress 1986) (Fig. S1A). Ambophily is also found in the genus *Salix,* some species of which are wind pollinated, while others are mostly bee pollinated (e.g., Ostaff et al. 2015) (Fig. S2B). Similarly, species of *Castanea* sometimes are wind pollinated (Regal 1982) and sometimes beetle-pollinated (Larue et al. 2021) (Fig. S2C). *Helwingia japonica* under some conditions may be ambophilous (Ao and Tobe 2015), while under others it is insect-pollinated only (Wu et al. 2020) (Fig. S2D). Dioecious *Sargentodoxa cuneata* (Fig. S2E) may be wind pollinated (Qin et al. 2007), but its female and male flowers are scented, with the anthers and pistils hidden within the pendulous petals, suggesting insect pollination. Pollination in the genus *Thalictrum* varies from anemophilous to insect pollinated (Abrahamczyk et al. 2023) (Fig. S2FG). Even normally wind pollinated families, such as Cyperaceae, included species that are sometimes insect-pollinated. Thus, *Cyperus obtusiflorus* and *C. sphaerocephalus* can be pollinated by bees, beetles, and flies (Wragg and Johnson 2011) and so are *Rhynchospora ciliata* and *Juncus allioides* (Costa and Machado 2012, Huang et al. 2013). *Juncus* species are particularly interesting in having pollen grains aggregated into tetrads (Fig. S1H) and many ovules per flower, traits that are typical of insect-pollinated species. Another example of possible anemophily is *Paris* species (Jacquemyn and Brys 2008; Fig. S2I). Although so far, no insect visits to flowers have been observed, its sticky pollen suggests insect pollination. To test whether a species is wind pollinated, we suggest shading the newly opened flowers or inflorescences in the field, to see whether airborne pollen grains are shed, besides the time-tested method of placing sticky glass slides downwind from the flowers to test for pollen deposition.

**References**

Abrahamczyk S, Struck JH, Weigend M. 2023. The best of two worlds: ecology and evolution of ambophilous plants. Biological Reviews, 98: 391-540.

Ao C, Tobe H. 2015. Floral morphology and embryology of *Helwingia* (Helwingiaceae, Aquifoliales): systematic and evolutionary implications. Journal of Plant Research 128: 161-175.

Costa ACG, Machado IC. 2012. Flowering dynamics and pollination system of the sedge *Rhynchospora ciliata* (Vahl) Kukenth (Cyperaceae): does ambophily enhance its reproductive success? Plant Biology 14: 881-887.

Cronquist A. 1981. An integrated system of classification of flowering plants. Columbia University Press, New York.

Endress, P. K. 1986. Floral structure, systematics, and phylogeny in Trochodendrales. Annals of the Missouri Botanical Garden, 73: 297-324.

Gan X, Cao L, Zhang X, Li H. 2013. Floral biology, breeding system and pollination ecology of an endangered tree *Tetracentron sinense* Oliv. (Trochodendraceae). Botanical Studies 54: 1-9.

Huang S-Q, Xiong Y-Z, Barrett SCH. 2013. Experimental evidence of insect pollination in Juncaceae, a primarily wind-pollinated family. International Journal of Plant Sciences 174: 1219-1228.

Jacquemyn H, Brys R. 2008. Density-dependent mating and reproductive assurance in the temperate forest herb *Paris quadrifolia* (Trilliaceae). American Journal of Botany 95: 294-298.

Larue C, Austruy E, Basset G, Petit RJ. 2021. Revisiting pollination mode in chestnut (*Castanea* spp.): an integrated approach. Botany Letters 168: 348-372.

Ostaff DP, Mosseler A, Johns RC, Javorek S, Klymko J, Ascher JS. 2015. Willows (*Salix* spp.) as pollen and nectar sources for sustaining fruit and berry pollinating insects. Canadian Journal of Plant Science 95: 505-516.

Qin HN, Zhou QY, Guo C. 2007. Reproductive biology of *Sargentodoxa cuneata* (Sargentodoxaceae). Acta Botanica Boreali-Occidentalia Sinica 27: 1980-1986.

Regal PJ. 1982. Pollination by wind and animals - ecology of geographic patterns. Annual Review of Ecology and Systematics 13: 497-524.

Wragg PD, Johnson SD. 2011. Transition from wind pollination to insect pollination in sedges: experimental evidence and functional traits. New Phytologist 191: 1128-1140.

Wu T, Tang J, Huang S-Q. 2020. Foraging behavior and pollination efficiency of generalist insects in an understory dioecious shrub *Helwingia japonica*. American Journal of Botany 107: 1274-1282.
